# Evaluation of *Dirofilaria immitis* antigen detection comparing heated and unheated serum in dogs with experimental heartworm infections

**DOI:** 10.1186/s13071-017-2445-5

**Published:** 2017-11-09

**Authors:** James Carmichael, Scott McCall, Utami DiCosty, Abdelmoneim Mansour, Linda Roycroft

**Affiliations:** 1 TRS Labs Inc., PO Box 5112, Athens, GA 30604 USA; 2 Statistical Consultant, Walton, KY USA

**Keywords:** *Dirofilaria immitis*, Heartworm, Antigen test, Heated serum

## Abstract

**Background:**

To evaluate whether heated serum allows for earlier detection of *Dirofilaria immitis* antigen, dogs with experimental *D. immitis* infections underwent weekly blood sampling to compare antigen results using both heated and unheated serum.

**Methods:**

One of two isolates (JYD-34 or Big Head™) were used to infect naïve laboratory beagle dogs. Serum was collected from dogs weekly and divided into two aliquots, heated and unheated. The samples designated as heated were placed in a heat block at 104 °C for 10 min then centrifuged with collection of the resulting supernatant. Two commercial ELISAs, DiroCHEK® (Synbiotics Corporation, Zoetis) and PetChek® (IDEXX Laboratories, Inc.), were used to conduct *D. immitis* antigen testing on all serum samples.

**Results:**

There was no statistical difference in the mean number of days from infection to positive *D. immitis* antigen status between the two commercial testing kits (DiroCHEK® versus PetChek®) with either heated or unheated serum. When unheated serum was utilized, very strong agreement between the two assays was demonstrated using Lin’s concordance correlation coefficient (*R*_*c*_ = 0.98). However, when heated serum was compared, Lin’s concordance correlation coefficient was only *R*_*c*_ = 0.64, showing a lesser agreement. There was a statistical difference in the mean number of days from infection to a positive test result for unheated serum when compared to mean days to positive status with heated serum. For DiroCHEK® the heated serum yielded a positive result 126.9 ± 18.9 days postinfection while the unheated serum yielded a positive result 162.6 ± 23.0 days postinfection; this was a significant 35.7 ± 32.2 days longer, on average, compared with heated serum. With PetChek® the heated serum yielded a positive result 131.5 ± 11.7 days postinfection while the unheated serum yielded a positive result 162.8 ± 23.8 days postinfection; this was a significant 31.3 ± 25.5 days longer, on average, compared with heated serum. The detection of *D. immitis* antigen earlier using heated serum was consistent for both heartworm isolates.

**Conclusion:**

Our results suggest heat treatment of serum may allow earlier detection of *D. immitis* antigen but with less consistency demonstrated across two testing platforms as compared with antigen detection using unheated serum.

## Background

Commercially available tests designed for detection of adult heartworm (*Dirofilaria immitis*) antigen in infected dogs are considered highly specific, with very good sensitivity and are widely used in the clinical setting [1–4]. The time between *D. immitis* infection and detection of antigen is suggested to be very consistent in infections at least 8 months old, inconsistent for infections 5 to 7 months old, and not detectable for infections of less than 5 months [1]. The period before adult heartworm antigen detection can be challenging for veterinary practitioners when beginning a patient on a prophylaxis program, especially during seasons when exposure to mosquito vectors may occur. A dog may potentially be infected with heartworm prior to beginning a prophylaxis program; but due to the prolonged maturation period, adult heartworm antigen is undetectable leading to a false-negative result. The American Heartworm Society currently recommends immediate adult antigen testing of a dog with unknown exposure history or noncompliant prophylaxis history and retesting 6 months later to rule out a prepatent infection [4].

A false-negative result with *D. immitis* adult ELISA antigen tests may also result from host-specific factors, such as highly bound antibody/antigen complexes. Antigen that is trapped within immune complexes likely eludes or lessens detection by an immunoassay thereby leading to an increase in false-negative results [5]. Recent studies utilizing pretreatment of serum samples with heat to disrupt immune complexes have demonstrated an increased sensitivity using commercial *D. immitis* antigen assays in dogs [6, 7] and cats [8]. The character of the immune response to parasite antigen likely varies between animals, affecting levels of free antigen available for detection in assays [5]. Antibody titers of *D. immitis*-infected dogs are noted to peak just prior to sexual maturation of the heartworm, suggesting initial antigen release might be complexed by available antibody [9]. It is possible that pretreatment of serum with heat would shorten the time until visual antigen detection using ELISAs in dogs with known heartworm infection dates, presumably by decreasing antigen blocking by host antibodies.

The primary objective of the current study was to evaluate the time until visual detection of *D. immitis* antigen using two commercial enzyme-linked immunosorbent assays (ELISA) – in naïve dogs with experimental heartworm infections – by comparing serum which was pretreated with heat to unheated serum. The known date of *D. immitis* infective larvae inoculation and sequential weekly blood sampling allowed for determination of the time (days) to antigen detection for heated and unheated serum in both ELISA tests. A secondary study objective was investigating whether time to detection of *D. immitis* antigen varied between two distinct heartworm isolates using heat treated or unheated serum.

## Methods

### Experimental infections

One of two distinct heartworm isolates was used to infect naïve laboratory beagle dogs in the current study. A description and background of each isolate follows. The JYD-34 isolate originated in a naturally infected dog from North Central Missouri (Keytesville). The dog also resided in Pittsfield, Illinois, prior to relocation to Keytesville. The exact geographic locale of the natural infection and the age of the infection are unknown. A blood sample was used to infect mosquitoes at TRS Labs Inc. in July 2010. The isolate has been maintained in dogs under strict laboratory conditions since that time and was validated by TRS Labs Inc. in April 2011 by antigen testing, microfilarial counts, and worm recovery. The JYD-34-infected dog used as a donor for propagation of infective third-stage larvae (L3) used in the current study was the first laboratory passage of the isolate.

The Big Head™ isolate originated in a naturally infected heartworm-positive Labrador retriever dog residing in Livonia, Louisiana. The duration of heartworm infection is unknown. A blood sample was used to infect mosquitoes at TRS Labs Inc. in August 2015. The isolate is being maintained in laboratory dogs under strict control and was validated in March 2016 by antigen testing, microfilarial counts, and worm recovery. The Big Head™-infected dog used as a donor for propagation of L3 for this study was the first laboratory passage of the isolate.

In the current study, seven naïve laboratory beagle dogs (males and females 6 or 7 months of age) were infected with the JYD-34 isolate; and a separate cohort of five naïve laboratory beagle dogs (males and females 8 months of age) were infected with Big Head™. All animals were inoculated subcutaneously in the inguinal area with L3 of *D. immitis* harvested from infected *Aedes aegypti* mosquitoes (Liverpool strain). Dogs were inoculated with a minimum of 35 infective L3. A 20-gauge needle was attached to a tuberculin syringe, and the contents of the syringe were injected into the animal subcutaneously in the inguinal area. The syringe was rinsed several times with Hanks’ Balanced Salt Solution and reinjected to ensure that all L3 were removed from the syringe. The syringe was then rinsed into a petri dish and examined for remaining L3. Any remaining L3 were injected into the animal as described.

### Blood sampling

A weekly blood sample was collected from the jugular vein of each dog into 8.5 mL vacuum tubes containing a serum separator plug. Blood was allowed to clot, and serum was separated by centrifugation. Serum was then divided into two aliquots and labeled, nonsystematically by an unmasked technician, with a number from a key corresponding to the dog and a designation as heated or unheated. In this way masking of the parasitologist interpreting the assay results was preserved. Weekly blood sampling of individual animals was discontinued when antigen-positive status was confirmed for both heated and unheated serum samples on two consecutive samplings on both assays. All dogs were housed in runs, either individually or pair-housed, indoors under controlled conditions at TRS Labs Inc. and cared for under the facility’s Program of Animal Care. The research protocol and all procedures were approved by the TRS Labs Inc. Institutional Animal Care and Use Committee.

### Antigen testing

Two commercial ELISAs in microtiter plate formats, DiroCHEK® (Synbiotics Corporation, Zoetis) and PetChek® (IDEXX Laboratories, Inc.), were used to conduct *D. immitis* antigen testing on all weekly serum samples according to the manufacturer’s instructions. Reported test performance for PetChek®: sensitivity 98% (95% CL 91.1–100%); specificity 100% (95% CL 95.5–100%) [10]. DiroCHEK® reported performance: sensitivity 100%; specificity 100% [11]. For each serum sample assigned as a heated aliquot, an additional processing step was performed prior to testing with the assays. The designated heated samples were placed in a heat block at 104 °C for 10 min then centrifuged (~14,000 rpm for 20 min), and the resulting supernatant was collected for testing with the assays. Heat treatment of designated aliquots and setup of the commercial assays was performed by an unmasked technician, while a masked parasitologist visually interpreted and recorded weekly assay results. A positive antigen test result, for each assay and serum type, was classified as “confirmed positive” once there were two consecutive weekly visual positive test results. The known date of infection allowed determination of the number of days to a confirmed positive result for unheated versus heated serum for each assay.

### Statistical methods

The individual animal was the experimental unit. All hypotheses were tested at a two-sided 0.05 level of significance. No animals were excluded from any analysis. To determine if the number of days to a confirmed positive test result differed between the heated and unheated serum, analyses were performed within each antigen test (DiroCHEK®, PetChek®), and within each serum type (unheated, heated); the paired t-test was utilized as the data was dependent within each subject. To measure the agreement between the two antigen tests when heated serum was used and when unheated serum was used, Lin’s concordance correlation coefficient was calculated. Analyses performed for testing the differences between each isolate (Big Head™, JYD-34) within both test and serum type utilized the t-test as the data were independent. Levene’s test was used to analyze homogeneity of the variances between the isolates within both test and serum type. All analyses were performed using R® software (R Core Team [2014]). R: A Language and Environment for Statistical Computing (R Project for Statistical Computing, Vienna, Austria; https://www.r-project.org/).

## Results

### Individual data

**Fig. 1:**
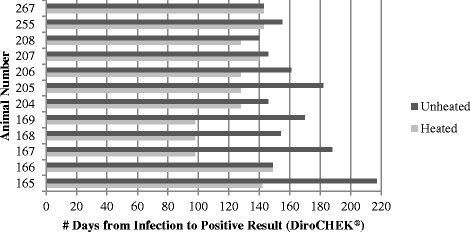
Number of days from infection to confirmed positive test result by individual animal and serum type (unheated versus heated serum) with the DiroCHEK® Antigen Test

**Fig. 2:**
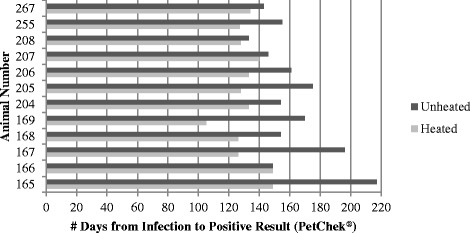
Number of days from infection to confirmed positive test result by individual animal and serum type (unheated versus heated serum) with the PetChek® Antigen Test

All 12 dogs experimentally infected with *D. immitis*, 5 infected with the Big Head™ isolate (animal numbers 165, 166, 167, 168, 169) and 7 infected with isolate JYD-34 (animal numbers 204, 205, 206, 207, 208, 255, 267), were diagnosed heartworm antigen positive with both DiroCHEK® (Fig. 1) and PetChek® (Fig. 2) assays, using both heated and unheated serum.

### Heated versus unheated serum by assay

Heat pretreatment of serum resulted in earlier detection of a positive antigen test from infection, on average, for both assays. For DiroCHEK®, mean time to confirmed positive was 126.9 (± 18.9) versus 162.6 (± 23.0) heated versus unheated, respectively (Table 1). For PetChek® mean time to confirmed positive was 131.5 (± 11.7) versus 162.8 (± 23.8) heated versus unheated, respectively (Table 1). For both the DiroCHEK® and PetChek® antigen tests there were significant differences in the mean number of days from infection to a confirmed positive test result when unheated serum was used compared with when heated serum was used. The unheated serum yielded positive results 35.7 (± 32.2); and 31.3 (± 25.5) days later, DiroCHEK® and PetChek® respectively, on average, compared to the heated serum (*P* = 0.0028 and *P* = 0.0014, respectively; Table 2).

**Table 1:** – Number of days from infection to confirmed positive test result

Antigen Test		Serum Tested^a^								
	Heated					Unheated				
	N	Mean (days)	SD^b^	Min	Max	N	Mean (days)	SD	Min	Max
DiroCHEK®	12	126.9	18.9	98	149	12	162.6	23.0	140	217
PetChek®	12	131.5	11.7	105	149	12	162.8	23.8	133	217

^a^Big Head™ and JYD-34 isolate data were combined for this table

^b^ *SD* standard deviation

**Table 2:** Difference in the number of days from infection to confirmed positive test result between the two serum types (unheated versus heated) within each antigen test

Antigen Test	Difference in # Days from Infection to Positive Result^a^ Unheated – Heated							*p*-value
	N	Mean (days)	SD^b^	Min	Max	95% CI^c^		
						**L** ^**d**^	**U** ^**d**^	
DiroCHEK®	12	35.7	32.2	0	90	15.2	56.2	0.0028*
PetChek®	12	31.3	25.5	0	70	15.0	47.5	0.0014*

*Statistically significant at *P* < 0.05

^a^Big Head™ and JYD-34 isolate data were combined for each analysis

^b^ *SD* standard deviation

^c^95% CI = 95% Confidence Interval around the mean difference

^d^ *L* lower bound of 95% confidence interval, *U* upper bound of 95% confidence interval

To determine if there was a difference between the DiroCHEK® and PetChek® antigen tests using heat-treated serum or unheated serum, a paired t-test was utilized to compare the difference in the number of days from infection to positive result (Table 3). Regardless of whether heated or unheated serum was used in the DiroCHEK® and PetChek® antigen tests, there was no significant difference in the mean number of days from infection to a confirmed positive result between the tests (*P* = 0.2405 and *P* = 0.9009, respectively; Table 3). On average, one test did not pick up on the dogs’ infection earlier than the other test.

**Table 3:** Difference in the number of days from infection to confirmed positive test result between the two antigen tests (PetChek® versus DiroCHEK®) within each serum type

Serum Tested	Difference in # Days from Infection to Positive Result^a^ PetChek® – DiroCHEK®							*p*-value
	N	Mean (days)	SD^b^	Min	Max	95% CI^c^		
						**L** ^**d**^	**U** ^**d**^	
Heated	12	4.6	12.8	˗16	28	˗3.5	12.7	0.2405
Unheated	12	0.2	4.5	˗7	8	˗2.7	3.0	0.9009

^a^Big Head™ and JYD-34 isolate data were combined for each analysis

^b^SD = standard deviation

^c^95% CI = 95% Confidence Interval around the mean difference

^d^L = lower bound of 95% confidence interval; U = upper bound of 95% confidence interval

Numerically the differences between the days from infection to positive test result comparing the two antigen tests when using unheated serum were smaller (mean = 0.2), with a tighter variance (± 4.5), from the differences between the days from infection to positive test result when using heated serum (mean = 4.6 ± 12.8) as the comparison. Lin’s concordance correlation coefficient was calculated to determine the measure of agreement between the two antigen tests when heated serum was used compared to when unheated serum was utilized.

**Fig. 3:**
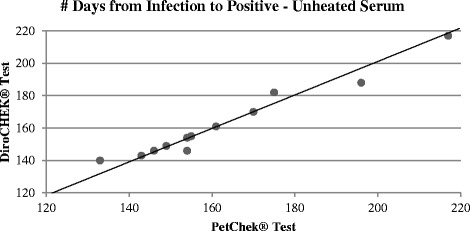
Scatter plot of number of days from infection to confirmed positive result for the PetChek® and DiroCHEK® Antigen Tests using unheated serum (with Lin’s correlation line y = x)

When unheated serum was utilized in both antigen tests, Lin’s concordance correlation coefficient was *R*_*c*_ = 0.98 (95% confidence interval = [0.94, 0.99]), showing a very strong agreement between the DiroCHEK® and PetChek® tests. This is visible in the tight clustering of the points around the y = x line in Fig. 3.

**Fig. 4:**
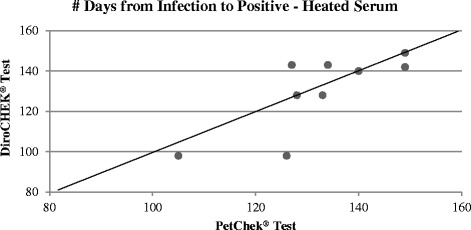
Scatter plot of number of days from infection to confirmed positive result for the PetChek® and DiroCHEK® Antigen Tests using heated serum (with Lin’s correlation line y = x)

When heated serum was utilized in both antigen tests, however, Lin’s concordance correlation coefficient was only *R*_*c*_ = 0.64 (95% confidence interval = [0.26, 0.85]), showing a lesser agreement between the DiroCHEK® and PetChek® tests than when the unheated serum was used. This is visible in the spread of the points around the y = x line in Fig. 4; they are not clustered around the y = x line as tightly as they are in Fig. 3. It should be noted there is overlap of three dogs leading to nine data points in Fig. 4.

### Big head™ versus JYD-34 isolate

**Fig. 5:**
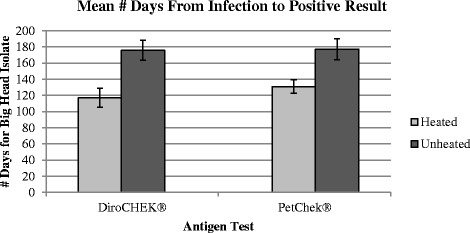
Mean number of days from infection to confirmed positive test result when heated versus unheated serum was used within each antigen test for dogs infected with the Big Head™ isolate

To investigate whether the type of isolate might respond differently when conducting antigen tests using heated versus unheated serum, analyses were performed within each isolate. For dogs infected with the Big Head™ heartworm isolate there was a significant difference in the mean number of days from infection to a confirmed positive test result when unheated serum was used compared to when heated serum was used (Fig. 5). The unheated serum yielded positive results 58.6 (± 34.9) days later, on average, compared with the heated serum when the DiroCHEK® antigen test was utilized (*P* = 0.0199), and 46.2 (± 31.1) days later, on average, compared with the heated serum when the PetChek® antigen test was utilized (*P* = 0.0292).

Table 4 depicts the results of a paired t-test demonstrating that neither the DiroCHEK® nor PetChek® antigen test detects a heartworm infection in a dog infected with the Big Head™ isolate sooner than the other. For dogs infected with the Big Head™ heartworm isolate and tested using heated and unheated serum, there was no difference between the assays in the mean number of days from infection to a confirmed positive test result.

**Table 4:** Difference in the number of days from infection to confirmed positive test result between the two antigen tests (PetChek® versus DiroCHEK®) within dogs infected with the Big Head™ heartworm isolate

Serum Tested	Difference in # Days from Infection to Positive Result^a^ PetChek® – DiroCHEK®							*p*-value
	N	Mean (days)	SD^b^	Min	Max	95% CI^c^		
						**L** ^**d**^	**U** ^**d**^	
Heated	5	14.0	13.1	0	28	˗2.3	20.3	0.0751
Unheated	5	1.6	3.6	0	8	˗2.8	6.0	0.3739

^a^Big Head™ isolate data only were used for this analysis

^b^ *SD* standard deviation

^c^95%CI = 95% Confidence Interval around the mean difference

^d^ *L* lower bound of 95% confidence interval, *U* upper bound of 95% confidence interval

**Fig. 6:**
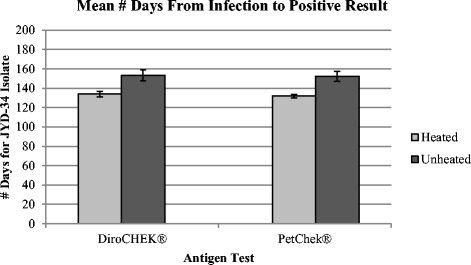
Mean number of days from infection to confirmed positive test result when heated versus unheated serum was used within each antigen test for dogs infected with the JYD-34 isolate

For dogs infected with the JYD-34 heartworm isolate there was a significant difference in the mean number of days from infection to a confirmed positive test result when unheated serum was compared with heated serum (Fig. 6). The unheated serum yielded positive results 19.3 (± 18.5) days later, on average, compared with the heated serum when the DiroCHEK® antigen test was utilized (*P* = 0.0328) and 20.6 (± 15.3) days later, on average, compared with the heated serum when the PetChek® antigen test was utilized (*P* = 0.0118).

Table 5 depicts the results of a paired t-test demonstrating that neither the DiroCHEK® or PetChek® antigen test detects a heartworm infection in a dog infected with the JYD-34 isolate sooner than the other. For dogs infected with the JYD-34 heartworm isolate and tested using heated and unheated serum, there was no difference between the assays in the mean number of days from infection to a confirmed positive test result.

**Table 5:** Difference in the number of days from infection to confirmed positive test result between the two antigen tests (PetChek® versus DiroCHEK®) within dogs infected with the JYD-34 heartworm isolate

Serum Tested	Difference in # Days from Infection to Positive Result^a^ PetChek® – DiroCHEK®							*p*-value
	N	Mean (days)	SD^b^	Min	Max	95% CI^c^		
						**L** ^**d**^	**U** ^**d**^	
Heated	7	˗2.1	7.7	˗16	5	˗9.3	5.0	0.4888
Unheated	7	˗0.9	5.1	˗7	8	˗5.6	3.9	0.6729

^a^Big Head™ isolate data only were used for this analysis

^b^ *SD* standard deviation

^c^95% CI = 95% Confidence Interval around the mean difference

^d^ *L* lower bound of 95% confidence interval, *U* upper bound of 95% confidence interval

## Discussion

Pretreatment of serum with heat generally resulted in earlier detection of *D. immitis* antigen, in both commercial heartworm antigen assays, when compared to testing unheated serum in experimentally infected dogs. It is of note that there was between-dog variability on the earliest visual detection with both heated serum and unheated serum. This variability is unlikely due to the number of adult heartworms as the fewest infective larvae inoculated, in any dog, was 35. TRS Labs Inc. historical rate of adult heartworm infection in dogs after an experimental inoculation of around 50 infective larvae, is greater than 50% (J. W. McCall, unpublished data, 2016), which is consistent with a published percent recovery of adult worms of 56% [3]. Therefore, in the current study it is likely that dogs had at least three female worms and, thus, a worm burden sufficient to allow antigen detection [1].

In experimental laboratory heartworm efficacy studies, a commonly accepted practice is to conduct antigen tests of presumably heartworm naïve laboratory animals, raised in mosquito-free indoor environments, just prior to inoculation with *D. immitis* infective larvae and again approximately 120 days postinfection. Current common convention is to use unheated serum in the antigen tests. If no antigen is detected in either test, the dogs are considered not to have been exposed to *D. immitis* prior to the experimental infection. In heartworm studies, any dog positive on an adult antigen test, either prior to experimental infection or 120 days post inoculation, is typically excluded from the study as previous exposure to *D. immitis* cannot be ruled out.

In the current study, the earliest confirmed positive using heated serum was 98 days and 105 days postinfection, DiroCHEK® and PetChek®, respectively. As noted, the time to positive status is important for studies with experimental *D. immitis* infections, as the negative antigen test 120 days post inoculation confirms a dog’s negative status at entry into the study. These data suggest if heartworm researchers choose to pretreat serum with heat prior to antigen testing dogs with experimental infections, testing at 120 days post infection may be too late to confirm a dog’s negative heartworm status at study entry. In the current study, without the availability to measure optical density readings, we used the second consecutive antigen positive date as the “confirmed positive,” meaning the earliest dog actually tested positive at 91 days after inoculation. If heated sera are to be used in experimental heartworm studies to confirm negative status of enrolled dogs, a post-inoculation date at less than 91 days should be considered. The data demonstrated the earliest confirmed positive with unheated serum was 140 and 133 days post infection, via DiroCHEK® and PetChek®, respectively, supporting the current 120-day testing convention used in heartworm studies to preclude early prepatent infections of dogs at enrollment.

It should be noted how closely the two commercial ELISA antigen assays were in agreement when unheated serum testing results were compared. As an example of the testing consistency, eight dogs tested positive at the same weekly sampling when unheated serum was tested. The remaining four dogs tested positive within one weekly sampling time. With heat treatment of serum, less agreement was demonstrated when the assays were compared. Four dogs tested positive on the same weekly sampling, five dogs demonstrated one weekly sampling difference, and two dogs tested positive four weekly samplings apart. Additional future work using other antigen testing platforms, lateral flow immunochromatographic or membrane-bound ELISAs, to establish repeatability of serum antigen detection before and after heat treatment should be pursued.

We considered whether experimental *D. immitis* isolate, Big Head™ versus JYD-34, may affect the time to antigen detection. Numerically, the mean difference between the time (days) to confirmed positive antigen status (unheated *minus* heated serum) from infection was smaller in dogs infected with the JYD-34 isolate (Figs. 5 and 6) with a lower SD in both assays. Although this difference in variance was not significant when analyzed using Levene’s test (*p*-values >0.05), this could be due to the small number of animals in both groups. Interestingly three dogs infected with the Big Head™ isolate (165, 167, 169) showed a difference of >65 days, and up to 90 days in one dog in one assay, between confirmed antigen-positive detection with heated serum compared with untreated. In comparison for the JYD-34 isolate, the largest difference between confirmed antigen-positive detection with heated serum compared with unheated serum, in any dog, was 54 days. This suggests some individual hosts may be induced to mount an immune response with highly bound antibody–antigen complexes (antigen blocking) with low levels of free antigen available for ELISA detection [5]. Of interest for future study in a larger sample of animals is whether the distinct heartworm isolate modulates production of more or less antibody–antigen complexes or factors that stimulate formation of these complexes.

As suggested in recent studies [6, 7] some canine serum samples may contain factors that inhibit *D. immitis* antigen detection, and heat treatment of the sample presumably frees the antigen making it available for detection. Dog 166 (Big Head™ isolate) was confirmed antigen-positive on the same day, using both assays, when unheated and heat treated serum were tested. On the DiroCHEK® assay, dog 267 (JYD-34 isolate) also tested positive on the same day with heated and unheated serum but demonstrated antigen blocking of 7 days when tested with PetChek®. The rest of the dogs in this study exhibited some duration of apparent antigen blocking, visible in Figs. 1 and 2 by the difference in lengths of the unheated and heated lines for each subject. Further investigation of *D. immitis* antigen blocking may elucidate whether an individual’s immune response – including the amount, specificity and type of response – leads to generation of blocking factors and whether the reaction is possibly modulated by parasite characteristics, including distinct isolates or exposure (experimental versus naturally infected).

## Conclusions

In this study, pretreatment of canine serum with heat allowed *D. immitis* antigen detection, on average, 35.7 ± 32.2 days sooner using DiroCHEK® and 31.3 ± 25.5 days sooner with PetChek® in dogs with experimental heartworm infections. Statistically, there was no difference in mean number of days from infection to a positive test result between DiroCHEK® and PetChek® with either heated or unheated canine serum; one test did not detect heartworm infection sooner. For experimental heartworm studies, the convention of antigen testing 120 days after experimental infection to exclude a preexisting heartworm infection was only supported when using unheated serum. Although this study is limited by small numbers of animals, especially related to comparing different isolate responses, the data generated from pretreatment of serum with heat raise interesting possibilities on exploring earlier heartworm detection.
